# Forgiveness in the Context of Divorce: A Cross-Cultural Measurement Invariance Study via Multiple-Group Factor Analysis (CFA) across Chile and Spain

**DOI:** 10.3390/ijerph18168236

**Published:** 2021-08-04

**Authors:** Diana Rivera-Ottenberger, Mónica Guzmán-González, Carlos Calderón, Sagrario Yárnoz-Yaben, Priscila Comino

**Affiliations:** 1 School of Psychology, Pontificia Universidad Católica de Chile, Santiago 7820436, Chile; dvrivera@uc.cl; 2 Millennium Institute for Research in Depression and Personality (MIDAP), Santiago 7820436, Chile; 3 School of Psychology, Universidad Católica del Norte, Antofagasta 1240000, Chile; ccalderon@ucn.cl; 4 Faculty of Psychology, Universidad del País Vasco-Euskal Herriko Unibertsitatea, 20018 Donostia, Spain; sagrario.yarnoz@ehu.es; 5 Education and Sport Faculty, Universidad del País Vasco-Euskal Herriko Unibertsitatea, 01006 Vitoria-Gasteiz, Spain; priscila.comino@ehu.eus

**Keywords:** divorce, forgiveness, measurement invariance

## Abstract

(1) Background: Current research on the factors involved in the adaptation process to divorce or separation has explored cross-cultural differences. An initial step in the cross-cultural field is to investigate whether the measurements applied are comparable in different cultural contexts. The aim of the present study is to test the measurement invariance of the Questionnaire of Forgiveness in Divorce-Separation (CPD-S); (2) Methods: The CPD-S was completed by 556 (M = 44.52, SD = 10.18) and 240 (M = 41.44, SD = 7.87) Chilean and Spanish divorced individuals, respectively. Confirmatory factor analyses in single samples and measurement invariance testing in a multi-group framework were conducted to test the cross-group equivalence; (3) Results: The single-factor structure of the CPD-S was supported in both countries. Measurement invariance analysis demonstrated that the CPD-S had partial scalar measurement invariance; (4) Conclusions: The evidence supports the conclusion that CPD-S operates similarly across both countries. Findings are discussed from a cross-cultural and methodological perspective.

## 1. Introduction

Divorce is a stressful experience that involves the reorganization of many areas of life [1], and is usually accompanied by adverse consequences on psychological well-being and mental health [2,3].

Previous studies on the process of adaptation to divorce or separation highlighted the importance of forgiveness, defined as a coping strategy that implies a decrease in negative feelings (i.e., resentment and anger) towards the offender and a reduced desire to punish him/her in response to transgressions [4,5,6].

Forgiveness is linked to a series of positive effects on psychological well-being, and mental and physical health, e.g., [7,8,9]. In the context of divorce, forgiveness is related to better adjustment to the separation, higher life satisfaction, and lower rates of post-breakup mental health problems [10,11,12,13,14,15,16]. Moreover, forgiveness of an ex-spouse promotes a positive co-parenting alliance [11], higher support from the ex-partner [17], and lessens negative consequences on the children [18]. 

In the examination of variations in the process of adaptation to divorce-separation and its associated factors, some studies have explored cross-cultural differences, e.g., [19,20,21]. An initial step of cross-cultural studies in this field is to establish whether the measures of the variables related to the adaptation to the divorce process operate similarly across cultures [22,23,24]. Hence, although a deeper cross-cultural examination of potential differences regarding divorce-relevant variables such as forgiveness is overdue, measurement equivalence should be established before an instrument is used for cross-cultural comparison. Measurement invariance means that the structure of the scale and the relationship between the observed indicators and their underlying latent variables are the same in different groups [25]. 

In order to contribute to fill this void, the aim of this study is to examine the cross-cultural equivalence of one of the most used scales for measuring forgiveness in the context of divorce, the Questionnaire of Forgiveness in Divorce-Separation (CPD-S) [6], in a sample of Chilean and Spanish divorced persons. 

The similarities between Chile and Spain provide a robust way to test CPD-S measurement invariance, considering that measurement equivalence is likely to be found in culturally similar countries [26]. Chile and Spain are predominantly Catholic countries, with a common historical and linguistic heritage. Legal divorce is relatively recent: divorce was legalized in Spain in 1981, and Chile was one of the last countries in the world to do so in 2004. Both countries place great value on the family and its preservation, and the two have shifted towards more positive attitudes regarding divorce [27,28]. Therefore, and considering these antecedents, we hypothesized that the CPD-S would exhibit measurement invariance, namely, that the CPD-S will operate similarly across Chile and Spain

The CPD-S was developed from the review of a series of instruments covering aspects of forgiveness that could be applied in the situation of divorce and separation. From an initial set of 15 items, 5 items were retained, covering aspects such as resentment, anger, blaming the other, and compassion. A brief five-item unidimensional scale was obtained. The analysis of the psychometric properties of the CPD-S reveals adequate reliability (Cronbach’s alpha = 0.78), and associations with measures of adaptation to separation, life satisfaction, willingness to co-parent, and the presence of behavioral problems in children [6]. Currently, this measure has been validated for its use in Chile [29] and Peru [30].

## 2. Materials and Methods

The study was approved by the Ethics Board of the institutions involved. Participants of the Spanish sample were contacted at various divorced and separated people associations, and at associations that provide support for separated families, such as Family Meeting Points in different provinces. Questionnaires were answered in these centers or at the parents’ home and mailed back anonymously to the research team using a prepaid envelope. 

For the Chilean sample, virtual and direct social networks were used to contact potential participants. The questionnaires were administered by trained assistants. In both cases, participants were provided with information about the objectives and nature of the study, emphasizing confidentiality, data security, and voluntary participation. Of the participants who completed the survey 1.3% returned incomplete questionnaires or inconsistent answers. Participants were 796 divorced or separated individuals, 240 (143 women and 97 men) from different Spanish communities and 556 (320 women and 236 men) from Chile. The mean age was higher in the Chilean sample (M = 44.52; SD = 10.18) than Spanish sample (M = 41.44; SD = 7.87); *t* (809) = 4.18, *p* = 0.000; Cohen’s *d* = 0.34. It was a well-educated group (in the Spanish sample, 45.8% had university studies; 43.5% in the Chilean sample). 

The length of the relationship was higher for the Chilean sample (M = 13.93, DS = 9.43) than for the Spanish group (M = 11.38; DS = 6.85), *t* (797) = 3.78, *p* < 0.05. The effect size was small (Cohen’s *d* = 0.31) The time since separation was similar in both groups, *t* (802) = 2.32, *p* = 0.07, Spanish (M = 49.00 months, DS = 49.25) and Chilean (M = 42.73, DS = 26.81). All participants in the Spanish sample (100%) reported having children, while the percentage was 85.80% for the Chilean sample.

### 2.1. Demographic and Background Information

Participants answered demographic questions (i.e., age, gender, country, educational level, and length of relationship) and provided divorce information (i.e., time since separation, presence and number of children, and custody). 

### 2.2. Forgiveness of the Former Partner

Forgiveness of the former partner was assessed with the 5-item Questionnaire of Forgiveness in Divorce-Separation (CPD-S; Yárnoz-Yaben and Comino, 2012). Each item is rated on a five-point Likert scale (1 = totally disagree; 5 = totally agree). Items are “I am angry toward my ex-partner”, “ I can’t help but blame my ex-spouse for causing the breakup”, “I have forgiven my ex-partner”, “Although my ex-partner’s behavior hurt me, I do not hold a grudge”, and “I hope my former spouse gets what he/she deserves for all the hurt he/she has caused me”(see Appendix A). The validated version was used for the Chilean sample [29]. The average score across the five items reflected the overall forgiveness of the ex-spouse. Higher scores indicated greater forgiveness. The scale consisted of a single factor and had a reliability score of 0.77 for the original version, and of 0.75 for the Chilean version. 

### 2.3. Data Analysis

The results are divided into two sections. The first presents the results of the one-factor model fit for the total sample. The second presents the results of the invariance analysis through a multi-group confirmatory factor analysis (MFCA). For the analysis of both models, we implemented the maximum likelihood with mean- and variance-adjusted (MLMV) estimation method, available in MPLUS v8 software (Muthén & Muthén, Los Angeles, USA. For the evaluation of the overall fit, we applied the most used indices. In addition to the goodness-of-fit index χ^2^, we considered the CFI (comparative fit index) and the TLI (Tucker–Lewis index). Values greater than 0.95 in both indexes are considered reasonable [31]. Finally, we considered root mean square error of approximation (RMSEA). Values lower than 0.06 are considered reasonable [32].

## 3. Results

### 3.1. Confirmatory Factor Analysis

Table 1 shows the results of the descriptive analyses of the items divided by sample. The results show that, in both samples, the item means remain around the midpoint of the scale (3 points). Additionally, when observing the kurtosis and skewness statistics, the scores do not deviate excessively from the normal distribution.

In light of these results, we decided to use the MLMV estimation method for the evaluation of the internal structure through CFA. This estimation method has been shown to be quite robust in the evaluation of small models (≤16 variables) and to non excessive violations of normality assumptions (kurtosis < 3).

**Figure 1:**
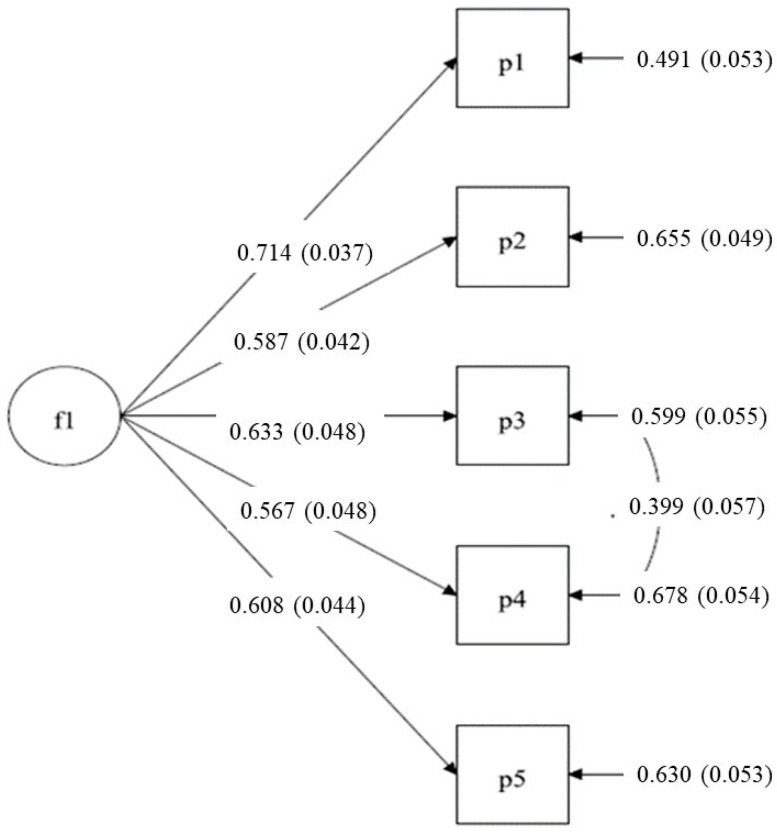
Standardized solution of final model.

The goodness-of-fit results of the one-factor model show a poor fit (χ^2^_5_ = 90.935; *p*-value = 0.000; CFI = 0.912; TLI = 0.823; RMSEA (IC 90%) = 0.147 (0.121–0.174)). However, the modification indices suggest the existence of a correlation between the measurement errors of items 3 and 4, which correspond to reversed items. By re-specifying the model leaving this parameter free to estimate, the fit indicators improve significantly (χ^2^_4_ = 12.168; *p*-value = 0.016; CFI = 0.992; TLI = 0.979; RMSEA = 0.051). These results suggest the presence of a method effect resulting from the wording item. Figure 1 shows the standardized solution of the final model.

### 3.2. Multi-Group Confirmatory Factor Analysis (MCFA)

Table 2 shows the results of the multi-group confirmatory factor analysis for the Chilean and Spanish samples at the different levels of invariance. Both the configurational level and the metric level (factor loading) obtain an excellent fit (CFI and TLI > 0.95; RMSEA < 0.06), with no significant differences between both levels (Δ*p*-value > 0.50). However, when restricting the intercepts between both samples to equality, the results show the presence of significant differences between both levels of invariance.

In order to detect the source of noninvariance, we have iteratively fitted a series of models that have been compared with the metric invariance level. In this way, we have identified a partial scalar invariance model, which shows that the source of noninvariance corresponds to the intercepts of items 1, 3, and 5 (ΔCFI, ΔTLI, and ΔRMSEA < 0.01) [33]. Although the presence of scalar invariance is desirable because it allows adequate comparison of means between groups, unfortunately very few cross-cultural studies manage to achieve this level of invariance [33]. 

## 4. Discussion

The goal of our study was to examine the measurement invariance of the CPD-S between Chilean and Spanish divorced individuals, given that testing the equivalence of instruments across groups is a prerequisite to draw valid conclusions in comparative studies. Our findings demonstrated that both groups attribute a similar meaning to the latent variable measured (i.e., forgiveness toward the ex-partner). 

Our results demonstrate that the CPD-S has the same latent structure in the two samples (a single-factor model), and showed partial scalar invariance, given that the level of some of the underlying items (intercepts) is different across groups. Three items (“I am angry with my ex-partner”, “I have forgiven my ex-partner”, and “I hope that my ex-partner gets what he/she deserves for all the harm he/she did to me”) were identified as noninvariant. Even when similarities between these two countries were anticipated, our results revealed that Spanish participants were more likely to manifest lower levels of anger and revenge desires, and higher levels of forgiveness than Chilean participants. A plausible explanation of these differing results is the predominance in Chile—but not in Spain—of a culture of honor, which mobilizes emotions such as anger and shame when a person’s reputation or social standing is in question [34]. In these cultures, separation is still perceived as a transgression to traditional social structures [35]. Of course, the analysis of deeper cultural currents and changes due to secularization is beyond the scope of this study, and a matter that deserves further interdisciplinary insights from fields such as cultural anthropology or the sociology of religions. 

However, it is worth noting that this type of result is frequent in cross-cultural studies. Achieving full measurement invariance is unlikely in practice, and cross-group comparisons can be examined with partial measurement invariance [36].

The remaining two items were invariant (“I can’t help but blame my ex-partner for causing the separation” and “Even though my ex-partner’s behavior hurt me, I do not hold a grudge”). Both Chilean and Spanish divorced individuals were inclined to endorse the same high level of blame to the ex-partner for the breakup, but at the same time, hold low levels of resentment towards him or her.

The current study has some limitations that need to be considered. First, the nonprobabilistic nature of the sample limits the generalization of the results. Second, different sampling procedures were implemented in the two sample groups, and well-educated divorced participants were more represented. 

To our knowledge, this is the first study that has explored the measurement equivalence of forgiveness in the specific context of divorce across two countries. Country differences in the mean level of a construct can be result of differences in understanding of certain concepts, or translation issues, and not testing the equivalence of a scale can prevent from detecting potential differences accurately. Our study is a first step in this line, providing empirical evidence on the equivalence of the forgiveness construct in two countries. Beyond the cross-cultural comparison, the relevant role played by forgiveness in the context of divorce could guide future research in the field, to offer useful insights for health care providers. 

## 5. Conclusions

The measurement invariance testing of the CPD-S supported the partial scalar invariance, which is sufficient to conclude the CPD-S operates in a similar way across both countries. Moreover, the results obtained highlight the relevance of examining measurement equivalence when conducting cross-cultural studies in general, and in the field of divorce research in particular.

## Figures and Tables

**Table 1:** Descriptive statistics and factor loading of confirmatory factor analysis.

	Spanish Sample					Chilean Sample				
	Mean	Standard Deviation	Asymmetry	Kurtosis	Factorial Loading	Media	Standard Deviation	Asymmetry	Kurtosis	Factorial Loadings
Item 1	2.950	1.410	0.152	−1.141	0.839	3.353	1.343	−0.344	−1.018	1.078
Item 2	3.225	1.489	−0.154	−1.365	0.697	3.248	1.286	−0.099	−1.022	0.856
Item 3	2.762	1.277	0.162	−0.811	0.746	3.272	1.230	−0.302	−0.771	0.750
Item 4	3.192	1.318	−0.159	−1.004	0.798	3.504	1.257	−0.590	−0.640	0.688
Item 5	3.454	1.384	−0.387	−1.015	0.860	3.923	1.260	−1.013	0.034	0.829

Note: All factor loadings are significative (*p* < 0.05).

**Table 2:** Descriptive statistics and factor loading of confirmatory factor analysis.

	Chi	df	*p*-Value	ΔChi	Δdf	Δ*p*-Value	CFI	TLI	RMSEA (IC 90%)
Configural	20.885	8	0.008				0.987	0.968	0.064 (0.031–0.098)
Metric	26.701	13	0.014	6.122	5	0.295	0.987	0.979	0.051 (0.023–0.079)
Scalar	65.343	18	0.000	41.392	5	0.000	0.954	0.948	0.081 (0.061–0.103)
Partial scalar (2,4) *	36.495	15	0.006	10.380	2	0.006	0.979	0.972	0.060 (0.035–0.085)

(*) The intercepts of items 2 and 4 are constrained to equals.

## Data Availability

Although the data are not publicly available due to privacy and ethical restrictions; it is available, on reasonable request, from the corresponding author.

## Appendix A

### Questionnaire of Forgiveness in Divorce-Separation

1. I am angry/angry toward my ex-partner. (R)
2. I can’t help but blame my ex-spouse for causing the breakup. (R)
3. I have forgiven my ex-partner.
4. Although my ex-partner’s behavior has hurt me, I do not hold a grudge.
5. I hope my ex-partner gets what he/she deserves for all the harm he/she did to me. (R)

Responses are given on a five-point scale: 1.—Strongly disagree; 2.—Disagree; 3.—Neither agree nor disagree; 4.—Agree; 5.—Strongly agree. (R) refers to reversed items.
